# Central white matter integrity alterations in 2-3-year-old children following prenatal alcohol exposure

**DOI:** 10.1016/j.drugalcdep.2021.108826

**Published:** 2021-08-01

**Authors:** Annerine Roos, Catherine J. Wedderburn, Jean-Paul Fouche, Sivenesi Subramoney, Shantanu H. Joshi, Roger P. Woods, Heather J. Zar, Katherine L. Narr, Dan J. Stein, Kirsten A. Donald

**Affiliations:** a SAMRC Unit on Risk and Resilience in Mental Disorders, Department of Psychiatry, Stellenbosch University, South Africa; b Department of Pediatrics and Child Health, University of Cape Town, South Africa; c Neuroscience Institute, University of Cape Town, South Africa; d Department of Clinical Research, London School of Hygiene & Tropical Medicine, United Kingdom; e Department of Psychiatry and Mental Health, University of Cape Town, South Africa; f Departments of Neurology and of Bioengineering, University of California, Los Angeles, USA; g Departments of Neurology and of Psychiatry and Biobehavioral Sciences, University of California, Los Angeles, USA

**Keywords:** Prenatal alcohol exposure, White matter integrity, Prenatal tobacco exposure, Development

## Abstract

**Background:**

Prenatal alcohol exposure (PAE) remains a potentially preventable, but pervasive risk factor to neurodevelopment. Yet, evidence is lacking on the impact of alcohol on brain development in toddlers. This study aimed to investigate the impact of PAE on brain white matter integrity in 2–3-year-old children.

**Methods:**

Children (n = 83, 30–37 months old) of the Drakenstein Child Health Study birth cohort, underwent diffusion MRI on a 3 T Siemens scanner during natural sleep. Parameters were extracted in children with PAE (n = 25, 56 % boys) and unexposed controls (n = 58, 62 % boys) using Tract-based Spatial Statistics, and compared by group. The contribution of maternal tobacco smoking to white matter differences was also explored.

**Results:**

Children with PAE had altered fractional anisotropy, radial diffusivity and axial diffusivity in brain stem, limbic and association tracts compared to unexposed controls. Notably lower fractional anisotropy was found in the uncinate fasciculus, and lower mean and radial diffusivity were found in the fornix stria terminalis and corticospinal tract (FDR corrected p < 0.05). There was a significant interaction effect of PAE and prenatal tobacco exposure which lowered mean, radial and axial diffusivity in the corticospinal tract significantly in the PAE group but not controls.

**Conclusion:**

Widespread altered white matter microstructural integrity at 2–3 years of age is consistent with findings in neonates in the same and other cohorts, indicating persistence of effects of PAE through early life. Findings also highlight that prenatal tobacco exposure impacts the association of PAE on white matter alterations, amplifying effects in tracts underlying motor function.

## Highlights

• Prenatal alcohol exposure alters white matter integrity in 2–3-year-old children.
• Effects of prenatal alcohol exposure on white matter integrity persist.
• Co-exposure of alcohol and tobacco amplify white matter alterations in motor tracts.

## 1 Introduction

Prenatal alcohol exposure (PAE) remains a potentially preventable, but pervasive risk factor to child brain development globally. The early years of life are a crucial time when risk factors, including toxic effects of alcohol exposure, may become embedded with lasting impact on neurodevelopment and function. Reviews on the effects of PAE on neurodevelopment in childhood suggest that there may not be a safe level of alcohol use during pregnancy (Charness et al., 2016; Dejong et al., 2019; Flak et al., 2014; Moreno, 2017). A meta-analysis by Flak and colleagues found that even light to moderate drinking is associated with behavioral problems in children with PAE between 9 months and 5 years (Flak et al., 2014).

Magnetic resonance imaging (MRI) has become an established approach to help understand structural, functional and metabolic alterations in the brains of children with PAE. Patterns of typical white matter development during infancy and early childhood have been well-established using MRI, specifically using diffusion tensor imaging (Geng et al., 2012; Hermoye et al., 2006; Huang et al., 2006; Lebel et al., 2019; Lebel and Deoni, 2018; Loh et al., 2012). White matter development and maturation is most rapid during the first year of life followed by a prolonged period of refinement into adolescence. However, by 2 years of age, many of the fundamental structural networks are in place. Commissural and projection tract connections of key motor, sensory and limbic regions are the first to be established, including the corpus callosum, cerebellar peduncles, corticospinal and thalamic tracts, and the fornix (Hermoye et al., 2006; Loh et al., 2012). Association tracts connecting frontal and temporal regions within hemispheres involved in cognition and behavior, such as the uncinate fasciculus, have a protracted period of maturation (Lebel and Deoni, 2018). Other major tracts such as the longitudinal fasciculi and the cingulum, connecting frontal, temporal and parietal regions also have prolonged developmental maturation into adulthood (Geng et al., 2012; Lebel et al., 2019).

Measures quantifying white matter development using diffusion tensor imaging (Lebel et al., 2019) include the standardized parameter of white matter integrity i.e. fractional anisotropy (FA) that generally increases with development. In turn, measures of diffusivity including perpendicular or radial diffusion (RD) toward myelin and axial diffusion (AD) along axons, decrease with development. Mean diffusivity (MD) refers to average diffusion that generally decreases with development, derived from the three eigenvalues of the diffusion tensor. Changes are most rapid in FA and RD during the first two years as myelination occurs and myelin matures, while alterations from the norm may suggest aberrant myelination and axon structure.

The majority of brain imaging studies of children with PAE have been conducted at school-age and adolescence. These studies have reported alterations in the structure and function of frontal, parietal, and temporal regions, in the cerebellum, limbic system and striatum, as well as in white matter tracts that connect these regions (Archibald et al., 2001; Lebel et al., 2012, 2008; Leigland et al., 2013; O’Leary-Moore et al., 2011; Sowell et al., 2008; Wozniak et al., 2006; Wozniak and Muetzel, 2011). In our setting, multimodal MRI and behavioral studies of young children have been safely and successfully conducted to investigate early neurodevelopment (Wedderburn et al., 2020). However, due to the acknowledged difficulties in acquiring MRI data in the pre-school group, very few reports of the impact of prenatal exposures on the developing brain, at age 2–3 years, have been published to date. This leaves a gap in our understanding of brain white matter microstructural development during this age. In addition, evidence is lacking on contributory effects of substances such as prenatal tobacco exposure on white matter integrity. Tobacco is commonly co-used with alcohol during pregnancy (Jarmasz et al., 2017; May et al., 2005; Vythilingum et al., 2012); and combined maternal use of alcohol and tobacco has been shown to worsen birth and health outcomes of children (Hamułka et al., 2018). Reviews report neurotoxic effects, adverse birth and health outcomes, and altered cognition in children following prenatal tobacco exposure (Gould et al., 2017; Scott-Goodwin et al., 2016). However, few studies have focused on the effects of combined use on brain structure.

The Drakenstein Child Health Study (DCHS) provides a unique opportunity to address these gaps in the literature. Prior work in this cohort found (Donald et al., 2018; Stein et al., 2015; Zar et al., 2015) altered white matter microstructural integrity in major association tracts (Donald et al., 2015) and disrupted functional connectivity between motor, somatosensory, striatal and brainstem/thalamic networks (Donald et al., 2016) in neonates with PAE. Another group investigating early effects of heavy PAE in an adjacent community, further reported diffusion differences in multiple tracts including projection, commissural and association tracts (Taylor et al., 2015). These findings are consistent with those seen in older cohorts, suggesting that the neurodevelopmental effects of PAE may be detected early in life. This study aimed to investigate white matter microstructural integrity in 2–3-year-old children with PAE in the DCHS birth cohort, thereby adding to our understanding of the impact of maternal substance use on white matter development during these crucial first years.

## 2 Methods

### 2.1 Study design and participants

**Fig. 1:**
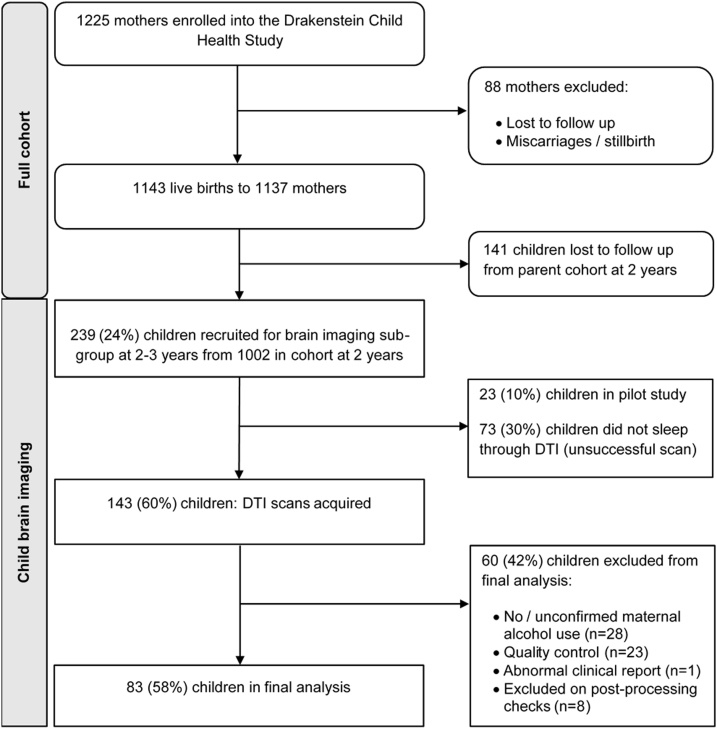
Flowchart outlining participation in the DCHS. Of the children for whom diffusion tensor imaging (DTI) was successful, 83 children were included in the final analysis.

The DCHS is a birth cohort study located in the peri-urban Drakenstein district of the Western Cape, South Africa, where prevalence of substance use is high (Stein et al., 2015; Zar et al., 2015). Public health service utilization is in excess of 90 % in these communities. Women aged 18 years or older were recruited at 20–28 week’s gestation while attending routine antenatal care at district clinics, while children born to them were followed from birth to track their lung health and neurodevelopment. A subset of mother-infant dyads participating in the DCHS were included in the brain imaging component. See Fig. 1 for a flowchart of brain imaging participation in relation to the larger DCHS cohort. Children were excluded if the mother had a positive urine screen for other drugs of abuse (e.g. methamphetamine, cocaine); if they were premature (< 36 weeks gestation); had low Apgar scores (< 7 at 5 min); significant neonatal complications, or genetic or congenital syndromes; or MRI contraindications including metallic and cochlear implants. Informed consent was obtained for participation in the main DCHS and from parents/caregivers of children participating in brain imaging studies. The study was approved by the University of Cape Town Health Sciences Faculty, Human Research Ethics Committee (Ref no 525/2012). The study was conducted according to the principles of the Declaration of Helsinki.

### 2.2 Maternal assessment

Mothers were followed from recruitment through pregnancy to delivery and mother infant pairs have subsequently been followed up to 6 years of age. Antenatal assessments included demographic and psychosocial measures, comprehensively detailed in previous publications on the cohort (Donald et al., 2018; Stein et al., 2015).

Alcohol use was assessed between 28–32 weeks’ gestation using the Alcohol, Smoking and Substance Involvement Screening Test (ASSIST) that has been validated by the World Health Organization (WHO) for use in this context (Humeniuk et al., 2008; Jackson et al., 2010). Children of mothers who scored > 10 on the ASSIST indicating moderate to high alcohol usage, were assigned to the PAE group. Mothers also provided retrospective information on frequency and quantity of alcohol use during any of the three trimesters according to levels defined by WHO. According to this data mothers also had to have used at least 2 drinks twice a week in any trimester to be included in the PAE group. Active tobacco smoking status during pregnancy was determined using maternal urine cotinine levels. This measure verified smoking status especially in cases of negative self-report that may occur due to stigma. Mothers with a cotinine level above 500 ng/mL were classified as active smokers.

### 2.3 Child assessment and imaging

Anthropometric measurements of children were collected during the imaging visit, and health information at birth were extracted from hospital records. Brain imaging of children was performed on a 3 T Siemens Skyra MRI scanner during natural sleep according to procedures described by Wedderburn et al. (Wedderburn et al., 2020). T1-weighted structural images were acquired with parameters: TR = 2530 ms; TE (1–4) = 1.69, 3.54, 5.39, 7.24; flip angle = 7°; slice thickness 1.0 mm; 176 slices; voxel size: 1.0 × 1.0 × 1.0 mm. Two diffusion-weighted images were acquired. One image was acquired in the anterior-posterior and another in a posterior-anterior phase direction, each with the following parameters: 30 diffusion directions; b-value 1 of 0 s/mm2; b-value 2 of 1000s/mm2; TR 7800 ms; TE 92 ms; slice thickness of 2 mm; voxel size: 1.8 × 1.8 × 2.0 mm.

### 2.4 Image processing

MRI data was checked for quality before preprocessing. Diffusion-weighted images had to have at least 15 usable volumes with minimal movement or other artefacts for inclusion. Preprocessing of diffusion tensor imaging data used TORTOISE version 3.1 (Tolerably Obsessive Registration and Tensor Optimization Indolent Software Ensemble) (Irfanoglu et al., 2017; Pierpaoli et al., 2010), implemented on the Centre for High Performance Computing (CHPC, Cape Town) cluster. TORTOISE was the method of choice compared to conventional diffusion processing pipelines given optimal performance for analyzing pediatric populations who are prone to move, due to rigorous correction and better anatomical registration ability (Taylor et al., 2016). Individual T1-weighted images provided an anatomical reference volume within TORTOISE and were inverted to have similar contrast to the diffusion b0 volume. The DIFF PREP module in TORTOISE was used to compute distortion corrections for participant motion, eddy currents and basic echo-planar imaging (EPI) distortions separately on each anterior-posterior and posterior-anterior encoded image. The DR BUDDI module (Irfanoglu et al., 2015) was used to merge encoded sets and perform further EPI distortion corrections.

FMRIB Software Library or FSL version 5 was used to perform diffusion tensor parameter fitting with the Tract-based Spatial Statistics (TBSS) pipeline, and to extract diffusion parameters based on anisotropic diffusion of water in tracts (Smith et al., 2006). TBSS is robust in quantifying such diffusion common to a specific cohort such as in 2–3-year-old brains that have foundational structural architecture in place (Gao et al., 2017). We used a study-specific template as recommended for studies investigating brains that are likely to differ from an adult MNI template and also for smaller studies, in order to enhance registration quality and optimize results (Bach et al., 2014; Smith et al., 2006). After preprocessing, FA images were created by applying brain extraction using BET and tensor extraction using DTIFIT (Smith, 2002). These images were then prepared using the first step of TBSS. In the next step, a representative template was created from our sample, registering it to FSL’s standard FMRIB58_FA template as an intermediate step, followed by linear registration of individual FA images into standard MNI space using FLIRT (Jenkinson et al., 2002) and mergence into one image. This merged template was then used as the registration target during the second step of TBSS. In the third step, a mean FA image and skeleton was derived. Finally, the mean FA skeleton was thresholded at 0.15 to create masks defining image voxels and a distance map for voxel-wise projection of individual FA images onto the mean FA skeleton. Subsequently the mean FA skeleton was used to map and derive MD, RD and AD images. Summary FA, MD, RD and AD values were extracted for 48 white matter tracts using the Johns Hopkins University ICBM-DTI-81 atlas (Mori et al., 2008).

### 2.5 Statistical analysis

Group differences in diffusion parameters were investigated in Statistica 13 using separate general linear models. Models controlled for sex and age of the child due to natural differences and associations in white matter diffusion (Geng et al., 2012; Long et al., 2018; Paolozza et al., 2017). In a model e.g. FA values of one tract were entered as the dependent variable, and group and sex (categorical variables), and age at scan (continuous variable) as relevant independent variables. Additional sociodemographic variables were identified a *priori* that differed significantly between groups. Thus, maternal tobacco use was included in subsequent models to assess for interaction effects of group and maternal tobacco exposure. Given the lack of prior evidence in this age group, and because multiple tracts are reportedly affected by PAE, this analysis was conducted using an exploratory whole brain approach. Further, because of potential functional significance at different developmental stages we grouped individual white matter tracts by tract type as association, brain stem, commissural, projection and limbic tracts (Mori et al., 2008). Results were corrected for multiple comparisons by tract type using the False Discovery Rate (FDR; q = 0.05). Association tracts included the sagittal stratum, external capsule, cingulum, fornix, superior longitudinal fasciculus, superior fronto-occipital fasciculus, and uncinate fasciculus. Brain stem tracts were the inferior, middle and superior cerebellar peduncle; medial lemniscus and corticospinal tract. Commissural tracts were the genu, body and splenium of the corpus callosum; and the tapetum. Projection tracts included the cerebral peduncle; anterior, posterior and retrolenticular parts of the internal capsule; pontine crossing; anterior, superior and posterior corona radiata; and posterior thalamic radiation. Limbic tracts were the fornix, fornix stria terminalis and uncinate fasciculus. Partial eta squared values are reported as an indication of effect size (Cohen, 1969; Richardson, 2011).

## 3 Results

The sample included 83 children of whom 25 had PAE and 58 children were unexposed healthy controls. Children had a mean age of 34 months (range 30–37 months). Groups had similar demographic and anthropometric details (Table 1). Maternal tobacco smoking was significantly higher in the PAE group compared to controls (p < 0.001).

**Table 1:** Demographic details of participants.

	PAE (n = 25)	Control (n = 58)	
	n (%) / mean (SD)		p
Child			
Sex, boys	14 (56 %)	36 (62 %)	0.604
Age, months	34.50 (1.94)	34.43 (1.58)	0.886
Gestation, weeks	39.36 (2.00)	39.24 (1.64)	0.777
Weight, kg	13.28 (1.77)	13.90 (1.73)	0.137
Head circumference, cm	49.26 (1.67)	49.80 (1.28)	0.112
Height, cm	91.39 (3.23)	91.50 (3.74)	0.914

Mother			
Education			0.476
Any tertiary	0 (0%)	4 (7%)	
Completed secondary	5 (20 %)	14 (24 %)	
Some secondary	16 (64 %)	39 (67 %)	
Primary	4 (16 %)	1 (2%)	
Income			0.504
<ZAR1000/month	8 (32 %)	17 (29 %)	
ZAR1000−5000/month	16 (64 %)	34 (59 %)	
>ZAR5000/month	1 (4%)	7 (12 %)	
Active tobacco smoking	18 (72 %)	18 (31 %)	<0.001
HIV infection	6 (24 %)	24 (41 %)	0.131

### 3.1 Group differences in white matter integrity following prenatal alcohol exposure

**Fig. 2:**
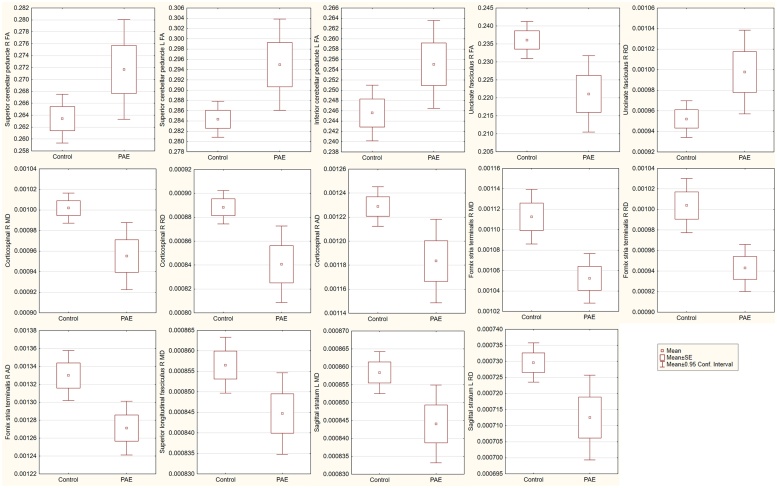
Boxplots of group effects (uncorrected). Patterns of effect were similar across tracts except for the uncinate fasciculus that had differences in the opposite direction. As indicated in the bottom right box, boxplots denote the mean parameter value with indication of the standard error (SE) and 0.95 confidence interval from the mean.

Table 2 present both uncorrected and corrected group differences in white matter integrity. Alterations were found in brain stem, association and limbic tracts, controlling for sex and age at scanning, between children with PAE and unexposed controls (Fig. 2). FA was higher in the cerebellar peduncles of children with PAE compared to controls with a small to medium effect size. FA was lower and RD higher in the right uncinate fasciculus with a medium effect size. MD, RD and AD were lower in the right corticospinal tract and fornix stria terminalis in the PAE group with a medium effect size. MD was also lower in the right superior longitudinal fasciculus with a small effect size. In addition, MD and RD were lower in the sagittal stratum in the PAE group compared to control children with medium effect sizes.

**Table 2:** Group differences in diffusion parameters by PAE status. There were also significant interaction effects of PAE with prenatal tobacco exposure in relation to most white matter parameters in the corticospinal tract.

				1Group		2Group		Group*tobacco	
Region	Hemisphere	Tract type	Effect in PAE	Partial eta^2^	p	Partial eta^2^	p	Partial eta^2^	p
Superior cerebellar peduncle	Right	Brain stem	↑ FA	0.0568*	0.032	0.0616**	0.027		ns
	Left		↑ FA	0.0907**	0.006	0.0902**	0.007		ns
Inferior cerebellar peduncle	Left	Brain stem	↑ FA	0.0553*	0.035	0.0508*	0.046		ns
Uncinate fasciculus	Right	Limbic#	↓ FA	0.0961**	0.005†	0.0766**	0.014		ns
			↑ RD	0.0665**	0.020	0.0497*	0.048		ns
Corticospinal	Right	Brain stem	↓ MD	0.1075**	0.003†	0.0334*	0.107	0.0777**	0.013
			↓ RD	0.1164**	0.002†	0.0397*	0.078	0.0717**	0.017
			↓ AD	0.0869**	0.008	0.0217*	0.195	0.0831**	0.010
Fornix stria terminalis	Right	Limbic#	↓ MD	0.0913**	0.006†	0.0600**	0.030		ns
			↓ RD	0.0972**	0.005†	0.0679**	0.020		ns
			↓ AD	0.0747**	0.014	0.0428*	0.067		ns
Superior longitudinal fasciculus	Right	Association	↓ MD	0.0499*	0.045	0.0186*	0.231		ns
Sagittal stratum	Left	Association	↓ MD	0.0728**	0.015	0.0337*	0.105		ns
			↓ RD	0.0842**	0.009	0.0355*	0.096		ns
									

Small, medium and large effect size as determined by partial eta^2^ respectively correspond to values of 0.0099, 0.0588, and 0.1379 (Cohen, 1969).

1 Model including sex and age at scanning.

2 Model including sex, age at scanning, and tobacco smoking.

* Small effect size.

** Medium effect size.

# Also association type.

† Survived FDR correction.

Group effects that survived correction were limbic tracts including FA in the right uncinate fasciculus (corrected p = 0.024); and MD (corrected p = 0.031) and RD (corrected p = 0.023) in the right fornix stria terminalis. Of the brain stem, MD (corrected p = 0.025) and RD (corrected p = 0.016) in the right corticospinal tract survived correction.

### 3.2 Contributing effects of prenatal tobacco exposure

**Fig. 3:**
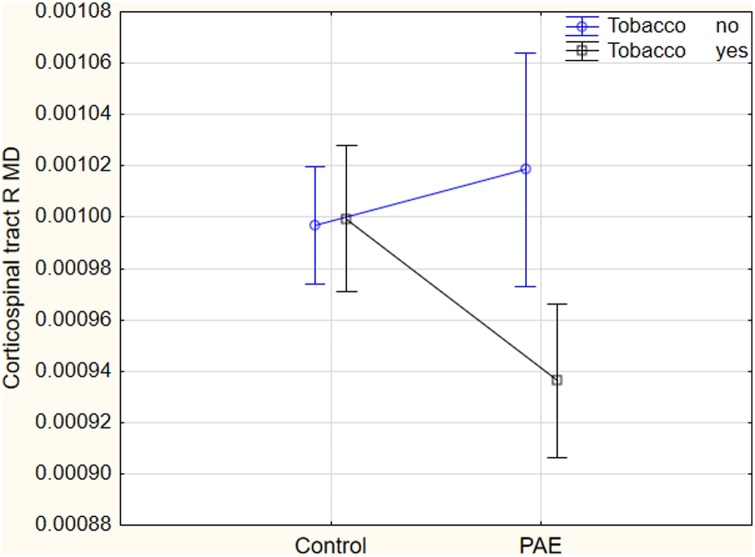
Interaction effect of alcohol and tobacco smoking on mean diffusivity in the corticospinal tract. Prenatal tobacco exposure lowered MD in the PAE group but not in controls. Vertical bars denote 0.95 confidence intervals.

Since there was a significantly higher proportion of mothers in the PAE group who smoked tobacco (72 %) compared to controls, smoking status was added to models to determine its contribution to group effects. Overall, prenatal tobacco exposure did not change the effect of PAE on FA in the cerebellar peduncles and uncinate fasciculus. However, findings in the right superior longitudinal fasciculus and left sagittal stratum were no longer significant after model adjustment. There were significant interaction effects of PAE and tobacco smoking in the right corticospinal tract with tobacco smoking lowering MD, RD and AD significantly in the PAE group but not in controls. To illustrate, the effect on MD is shown in Fig. 3. Overall, adding prenatal tobacco exposure to the models attenuated the findings. None of the findings survived correction after adding tobacco exposure as a covariate. The interaction effect suggest that prenatal tobacco exposure is an important contributor to diffusivity differences in the PAE group.

## 4 Discussion

This study demonstrates widespread alterations in white matter microstructural integrity in children with PAE at 2–3 years of age. These differences are consistent with alterations seen in neonates suggesting that they persist from birth. The results also reveal that prenatal tobacco exposure may be an important contributor to white matter differences alongside alcohol exposure in motor tracts in this population.

### 4.1 Effects of prenatal alcohol exposure

The findings reported in this study map onto brain stem, limbic, and association tracts commonly reported to have altered white matter integrity in older children with PAE. These tracts include the cerebellar peduncles, uncinate fasciculus, corticospinal tract and longitudinal fasciculi (see comprehensive review by (Ghazi Sherbaf et al., 2019)). They underlie a range of cognitive, behavioral and emotional functions. These include executive function, memory, decision-making, and emotional regulation. Further, although we did not find involvement of the corpus callosum, we did find lower diffusion in the fornix stria terminalis (posterior midline portion of the fornix). This tract is located adjacent to the splenium of the corpus callosum that has been most consistently reported to be affected by PAE in other studies (Ghazi Sherbaf et al., 2019). These two tracts develop together and reach maturity during a similar window during the first two years of life, and may thus show similar compensatory development after PAE (Hermoye et al., 2006; Jacobson et al., 2017; Loh et al., 2012). Both hypoplasia of the corpus callosum and dysplasia of the fornix have been independently associated with PAE in older children, supporting this view (Boronat et al., 2017). The fornix stria terminalis extends from the limbic system to cortical regions and microstructural alterations in this tract have been associated with externalizing behavior in typically developing children (Andre et al., 2020). Nevertheless, the finding of differences in the region of the fornix should be viewed as exploratory as TBSS is limited in its anatomical registration of the fornix in relation to adjacent fibers (Bach et al., 2014). Future studies may consider using additional techniques such as deformable registration of diffusion tensor images to optimize explicit fiber orientation (Bach et al., 2014; Zhang et al., 2006).

Similarly, altered FA in the uncinate fasciculus has also been associated with externalizing and internalizing behaviors in typically developing children which may underlie risk for emotional and behavioral dysregulation (Andre et al., 2020). The uncinate fasciculus connects temporal (including the amygdala) to inferior frontal brain regions and was shown to have lower FA and higher RD in children with PAE in our cohort. White matter integrity of the uncinate fasciculus has further been associated with learning, memory, attention and language in children (Olson et al., 2015; Von Der Heide et al., 2013; Walton et al., 2018). These are functional outcomes that have been well described as being affected in children with PAE.

Our findings at 2–3 years of age support the hypothesis that the microstructural alterations in major tracts may represent persistent effects of PAE on the early trajectory of brain development. Our findings in this study are consistent with results from the DCHS imaging cohort as neonates. There is some overlap in the children scanned as neonates and at 2–3 years. In particular, the neonate findings included altered white matter integrity in the inferior cerebellar peduncle and the superior longitudinal fasciculus (Donald et al., 2015), and disrupted functional connectivity of motor, somatosensory, striatal and brainstem/thalamic networks (Donald et al., 2016). Given that structural and functional networks develop in an integrated manner, we expect that the altered white matter microstructure observed here may associate with functional differences. In agreement, another study in neonates with PAE (Taylor et al., 2015) reported similarly altered white matter integrity to those in our sample of 2–3-year-olds, with differences in brain stem and association tracts. However, since our findings in the superior longitudinal fasciculus and sagittal stratum (includes the inferior longitudinal fasciculus and fronto-occipital fasciculus) were no longer significant after adjustment of models that included tobacco exposure, the separate contributing effects of prenatal alcohol and tobacco exposure is important to consider.

The direction of findings in the different tracts at this age needs to be explored in the context of what is known about the developmental trajectory and maturation of white matter. Typically, development in white matter is believed to be represented by increasing FA and decreasing MD, RD and AD. However, there are some exceptions e.g. the uncinate fasciculus shows variable patterns (Dean et al., 2017; Loh et al., 2012) as was evidenced by lower FA and higher RD in this tract in this cohort. However, in the context of prenatal insults or delayed maturation an altered pattern may be expected, although this may manifest differently at different ages in different tracts. We interpreted our current findings against the described typical direction suggesting either optimal white matter maturational development or impairment at this age. In this context, for the children with PAE, findings would be expected to occur in the direction suggesting white matter impairment. Altered RD and AD (contributing to MD) and the standardized FA metric, respectively, suggest changes in myelination, and axon development or density. This provide clues to how microstructure may have been affected in both the corticospinal tract and fornix stria terminalis in our 2–3-year-old group with PAE. Thus, in agreement with our findings, higher anisotropy and lower diffusivity likely reflecting values outside known or optimal ranges for this age, suggest accelerated phases of neural maturation (Fryer et al., 2009; Taylor et al., 2015). These findings may also suggest prolonged maturation in children with PAE that may prevent optimal network specialization and development. Similarly, such patterns have been found in children with a familial history of alcohol abuse that may precede future behavioral and cognitive problems, and risk for substance abuse (Cservenka, 2016; Squeglia et al., 2014). Researchers have also described the impact of PAE on developing microstructure with variable patterns and directions of effect (Lebel et al., 2008; Wozniak and Muetzel, 2011). Other factors suggested to affect white matter structure include specific damage to crossing fibers, or additionally through different mechanisms, tobacco smoking or other drugs of abuse (Lebel et al., 2008).

### 4.2 Effects of co-exposure to alcohol and tobacco

Notably prenatal tobacco exposure impacted the association of PAE on white matter integrity, with lower diffusion in the corticospinal tract of the PAE group after model adjustment. Generally, it is difficult to tease out contributing effects of substances on fetal brain development. Substances are often used together. Different substances may affect similar neural systems, but not in the same way. For instance alcohol, tobacco, cocaine and cannabis all associate with epigenetic dysregulation and exert effects on protein receptors and transporters, but with differing bodily and functional manifestations (Scott-Goodwin et al., 2016). Nevertheless, this cohort was screened for illicit substance use (together using the ASSIST and through urine screening in pregnancy). While we acknowledge that both of these screening measures have their limitations, this adds further validity to our findings, removing the potential confounding from maternal illicit drug use. Thus, prenatal exposure to both alcohol and tobacco may significantly impact tracts underlying motor development, and specifically myelination (i.e. RD) and axon development (i.e. AD).

The corticospinal tract is key to motor function extending into the primary motor, premotor and supplementary, and somatosensory cortices. Limited evidence in infants show that prenatal tobacco exposure alters structural volumes and white matter microstructure (Pulli et al., 2019), and that it changes brain activity in neonates exposed to tobacco or alcohol (Shuffrey et al., 2020). Specifically, prenatal tobacco exposure was the main prenatal risk factor driving smaller cerebellar vermis size in children with PAE (Hemingway et al., 2020). The corticospinal tract interconnects the cortex with the cerebellum (Jellison et al., 2004), of which the vermis is involved in aspects of motor function including motion and posture. This suggests that it is crucial to consider together the effects of alcohol and tobacco on brain development and specifically motor function. More studies are needed in this area.

### 4.3 Limitations and strengths

As expected in an imaging study in this age group, sample size was reduced by movement and other technical artefacts. This is a common occurrence when imaging young children who are prone to move, introducing potential selection bias. However, demographic and developmental profiles were similar between those whose imaging was successful and those for whom we failed to achieve usable diffusion tensor imaging data (see (Wedderburn et al., 2020)). Secondly, the approach we chose to take in this particular study was exploratory. The rationale for this is based on the fact that while there is a reasonable quantity of data on the impact of PAE on white matter integrity in older children, there is a lack of evidence in young children. Nevertheless, the majority of our findings were of medium effect size and consistent with previous findings from other cohorts as well as with our own findings in this cohort at an earlier time point. Longitudinal changes into older childhood on white matter microstructure following PAE remain to be explored. The separate effects of prenatal alcohol and tobacco exposure should be confirmed in larger samples of young children. Thirdly, although TBSS is widely used and robust in its anatomical specificity it has limitations. The method cannot distinguish the crossing and directionality of fibers, the quality of image registration may be inadequate, the meaning of findings is not exact, and different parameter settings across sites may bias results (Bach et al., 2014). Finally, the potential contribution of confounders such as early adversity, environment and psychosocial variables to microstructural alterations following PAE, need be investigated in larger samples.

## 5 Conclusion

This study demonstrates altered white matter microstructural integrity observable at 2–3 years of age after PAE. The findings are consistent with previous work in neonates, indicating persistence of effects that have potential to impact cognition and behavior over time. A key novel finding is that prenatal tobacco exposure amplified effects in tracts underlying motor function in PAE. These findings have clinical implications for the focus of intervention strategies given that mothers who drink alcohol during pregnancy often also report tobacco smoking. Evidence on both alcohol and tobacco use during pregnancy indicate toxic neural effects on the fetus and adverse child outcomes.

## Contributors

DJS, HJZ and KAD designed the study. KLN, SHJ and RPW collaborated in design of MRI components and data management. AR led the write-up. CJW and SS were involved in scanning procedures. AR, JF and CJW were involved in data management and analysis. All authors provided critical input on the paper and approved the final version of the paper.

## Funding

The study was funded by the 10.13039/100000865Bill and Melinda Gates Foundation (OPP 1017641). Additional funding was provided by the SA Medical Research Council, 10.13039/501100001321National Research Foundation, Academy of Medical Sciences Newton Advanced Fellowship (NAF002/1001) funded by the UK Government’s Newton Fund, by 10.13039/100000027NIAAA via (R21AA023887), by the Collaborative Initiative on Fetal Alcohol Spectrum Disorders (CIFASD) developmental grant (U24 AA014811), by a US Brain and Behavior Foundation Independent Investigator grant (24467), and by a Wellcome Trust Research Training grant (203525/Z/16/Z).

## Data availability

Data are available from the authors upon reasonable request as per cohort guidelines.

## Declaration of Competing Interest

No conflict declared.
